# Wood Derived Fast Pyrolysis Bio-liquids as Co-feed in a Fluid Catalytic Cracking Pilot Plant: Effect of Hydrotreatment on Process Performance and Gasoline Quality

**DOI:** 10.1021/acs.energyfuels.2c01736

**Published:** 2022-08-10

**Authors:** Helene Lutz, Marco Büchele, Florian Knaus, Alexander Reichhold, Wolfgang Vollenhofer, Robbie Venderbosch

**Affiliations:** † Institute of Environmental, Chemical and Bioscience Engineering (ICEBE), Technische Universität Wien, Wien 1060, Austria; ‡ OMV Refining & Marketing AG, Trabrennstraße 6−8, 1020 Vienna, Austria; § BTG Biomass technology Group B.V., Enschede 7521, The Netherlands

## Abstract

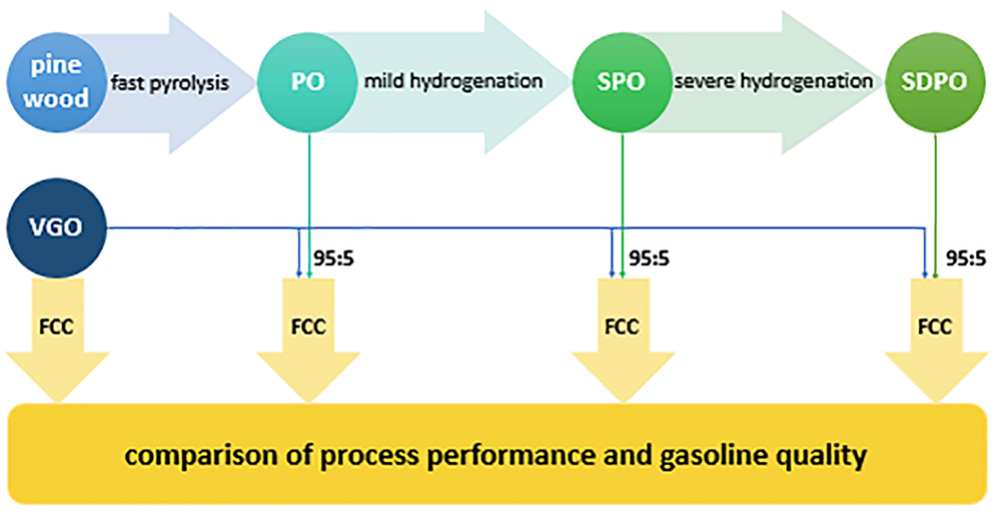

Co-feeding biogenic feeds in fluid catalytic cracking (FCC) units benefits from exploiting existing refinery assets to produce biogenic fuels. It is the most cost-effective way to comply with step-by-step increasing the target of renewable energy in road and rail transport of the European Union. Fast pyrolysis bio-liquids derived from wood offer a unique opportunity to reach those targets without having to address the typical food vs fuel debate. In the present work bio-liquids derived from pinewood in different stages of treatment were tested for their processability in a pilot scale fluid catalytic cracking plant at 550 °C. Specific focus is on the quality of the derived gasoline fractions. All samples were co-fed with vacuum gas oil, a typical FCC feed. Relevant parameters to qualify the produced gasoline as blending component were analyzed. As main results, none of the parameters examined significantly affect the quality of the—now partially biogenic—gasolines, demonstrating the potentiality of the co-FCC process as a possible near future pathway to ensure high biofuel contents in commercially available fuels.

## Introduction

To comply with the step-by-step increasing minimum target of the share of renewable energy within the final consumption of energy in the transport sector set by the European Union (EU), the commercialization of new biofuel production technology will be necessary. In 2019, 74 million tons of gasoline were consumed by the EU Member States,^1^ while the globally consumed amount of liquid fuels estimates 97.4 million barrels per day in April 2022.^2^ By 2030, the share of renewable energy must reach a minimum share of 14% in each Member State.^3^ The package of proposals called “Fit for 55: delivering the EU’s 2030 Climate Target on the way to climate neutrality” even states that a 90% reduction in overall transport emissions by 2050 is required to reach the goal of climate neutrality.^3^

The Project “WASTE2ROAD–Biofuels from Waste to Road Transport” funded by the European Framework Programme for Research and Innovation Horizon 2020, aims to establish cost-effective value chains from low-cost biogenic residues and waste fractions to biofuels. The objective is to reach overall biomass-to-fuel-carbon yields exceeding 45% while reducing greenhouse gases emissions by more than 80%. This may be done by using existing refinery technologies to allow a quick and easy implementation. The project partners cover the whole value chain from waste management to tests of the created biofuels.^4^

The fluid catalytic cracking (FCC) process plays a key role in integrated refinery as one of the primary conversion processes. FCC units convert high-boiling petroleum fractions into lighter products, which are usually products of higher value,^5^ such as gasoline and olefins. The influence of the reaction temperature^6^ and the used catalyst^7^ on the product spectrum and the deployable feeds make it a process with a high degree of tailorability.^8^

The co-feeding of biomass-derived oils^9,10^ such as vegetable oils,^11^ fatty acid methyl^12^ ester (FAME), or pyrolysis oil from biomass^13−16^ as well as the use of waste-derived pyrolysis oils^17,18^ in FCC units has generated a lot of attention in the scientific search for possibilities to provide fuels with smaller carbon footprints. Some of these alternative feeds are targets of the food vs fuel dilemma, whereas others use educts currently declared as waste. The next step on the path of implementing this research in the industrial setting is to verify the usability of the produced FCC gasoline as a gasoline blending component.

## Experimental Section

### Materials and Methods

#### Bio-liquids

**Figure 1:**
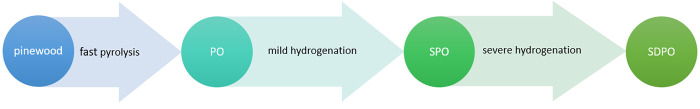
Schematic of the processing steps from pinewood to stabilized deoxygenated pyrolysis oil.

The bio-liquids were provided by Biomass Technology Group B. V. (Netherlands; BTG), and their properties are listed in Table 2. The utilized bio-liquid batches were taken between the different processing steps pinewood undergoes during its conversion to so-called stabilized deoxygenated pyrolysis oil. A schematic of the process is provided in Figure 1. Details to the different conditions of the applied hydrotreatments are listed in Table 1. The pinewood is processed via fast pyrolysis to pyrolysis oil (PO), which is hydrogenated at mild conditions to stabilized pyrolysis oil (SPO) over a dedicated Picula catalyst. The SPO is further hydrogenated under more extreme conditions using conventional catalysts to so-called stabilized deoxygenated pyrolysis oil (SDPO).

**Table 1:** Conditions of the Hydrotreatments

	catalyst	temp (°C)	pressure (bar)
mild hydrogenation	Picula	80–250	200
severe hydrogenation	CoMo/NiMo	>300	200

#### Vacuum Gas Oil and Catalyst

Both the hydrotreated vacuum gas oil (VGO) and the equilibrium catalyst were provided by the OMV Schwechat refinery.

Hydrogenation of VGO aims to remove impurities, such as nitrogen and sulfur.^19^ In Table 2 different properties of the hydrogenated VGO provided by OMV are listed in comparison to the properties provided by BTG for the bio-liquids.

**Table 2:** Comparison of the Properties from the Used Feeds^a^

param	unit	PO	SPO	SDPO	VGO
total sulfur	mg/kg	n.d.	n.d.	n.d.	291
total nitrogen	mg/kg	n.d.	n.d.	n.d.	275
nickel	mg/kg	<1	n.d.	n.d.	2
vanadium	mg/kg	<1	n.d.	n.d.	2
carbon	wt %	43.9	54.1	85.5	n.d.
hydrogen	wt %	7.9	9.3	10.4	n.d.
water content	wt %	20.5	8.9	0.5	n.d.
acid no.	mg of KOH/g	n.d.	35.2	7.0	n.d.
pH		n.d.	5.29	3.83	n.d.
ash content	wt %	0.01	n.d.	n.d.	n.d.
Conradson carbon residue	wt %	20.459	10.780	1.973	0.202
total aromatics	wt %	n.d.	n.d.	n.d.	23.8

a n.d., not determined.

Compared to fresh catalyst, the use of equilibrium catalyst provides a proper simulation of industrial plant conditions within a pilot plant.^20^ The provided equilibrium catalyst consists of two commercially available zeolite catalysts designed to maximize the production of propylene, butanes, and butenes.

#### Pilot Plant

**Figure 2:**
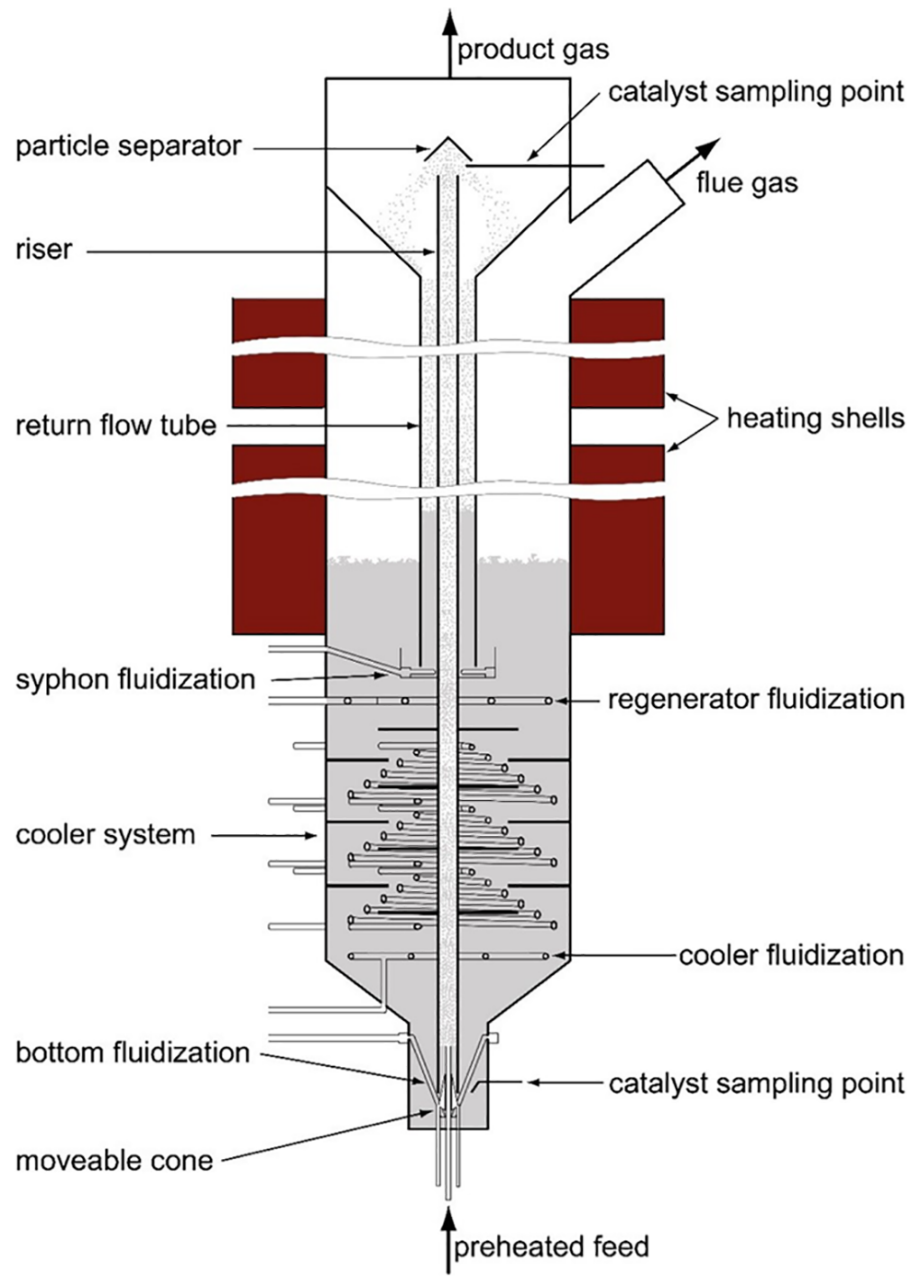
Schematic of the FCC pilot plant.

The FCC pilot plant, situated at the Institute of Chemical, Environmental and Bioscience Engineering (ICEBE) at Technical University Wien, was developed by Bielansky,^21^ based on a prototype designed by Reichhold.^22^ The continuously operating pilot plant is constructed as an internally circulating fluidized bed system, where the reactor section (riser) is located in the regenerator section. In Figure 2 a schematic of the pilot plant is depicted.

Feeds are pumped into a tubular oven to be preheated close to boiling temperature. This occurs via a feeding system that allows the use of two different pumps, enabling the co-feeding of two feeds, if these are not miscible. In the case of miscible feeds, one of the pumps can be bypassed. After the preheating, the feedstock is injected into the riser to come into contact with the hot catalyst. This leads to instant evaporation and thus an increase in volume by several orders of magnitude, creating an upward flow that transports the catalyst particles from the bottom of the plant through the riser top where they are separated from the product gas and fall down into a siphon. This siphon and the bottom section (so-called bottom) act as a gas barrier between the riser and the regenerator. The siphon also serves as a stripper that removes the remaining product gas from the catalyst particles. The product gas is transported to a flare or sampled to carry out further analysis. Oxygen, carbon monoxide and carbon dioxide are measured online.

Catalyst particles enter the regenerator through the siphon, where the coke deposited on the catalyst particles during the cracking process in the riser is combusted at a temperature of 610 °C. The combustion of coke ensures the regeneration of the catalyst and provides the thermal energy that is needed to facilitate the endothermic cracking reactions. The hot catalyst then passes through the cooler section where heat exchangers, operated with air or water, are applied to control the temperature of the catalyst that enters the bottom.

Bottom, siphon, and riser of the pilot plant are fluidized with nitrogen since the catalytic cracking reaction has to take place in the absence of oxygen, whereas cooler, regenerator, and free board sections of the pilot plant are fluidized with air to feed the oxygen required in the combustion reactions. Key data of the pilot plant are listed in Table 3.

**Table 3:** Key Data of the FCC Pilot Plant

total height	3.2 m
riser length	2.5 m
riser diameter	0.0215 m
regenerator diameter	0.33 m
regenerator temperature	500–800 °C
riser temperature	400–700 °C
pressure	atmospheric
catalyst mass	45–75 kg
feed rate	1.5–8 kg/h
riser residence time	∼1 s
catalyst circulation rate	0.5–5 kg/min
C/O ratio	10–50

During the sampling process, part of the product gas is directed thru a system of coolers (water and ethanol (−15 °C)) to condense all products that are liquid at room temperature. Afterward the gashouse phase passes through a gas collection tube and a gas meter. Each sample collection takes place over the course of 15 min. Throughout an experiment several samples are collected, which are analyzed multiple times.

The experiments were conducted at a feed temperature of 320 °C, a riser temperature of 550 °C and a feed rate of 2 kg/h, resulting in a C/O ratio between 23 and 27. The experimental ID’s and compositions are described in Table 4.

**Table 4:** Experiment Parameters and ID’s

expt ID	VGO content of feed (wt %)	admixture	admixture content of feed [wt %]
PO	95	PO	5
SPO	95	SPO	5
SDPO	95	SDPO	5
**VGO**	100		

#### Analysis

A so-called lump model is used to analyze the data. In this model, the products are categorized in different groups/lumps, which are characterized as a group, eliminating the need of characterizing every occurring component. The lump model is further detailed in Table 5.

**Table 5:** Details of the Emplyed Lump Model and Used Analysis Methods

fraction	product group	boiling range, composition	analysis method
gaseous	carbon oxides	CO, CO_2_	nondispersive infrared spectroscopy
	hydrocarbon gas	C1–C4	gas chromatography
liquid	gasoline	<210 °C	simulated distillation, further measurements
	light cycle oil	210–320 °C	simulated distillation
	residue	>320 °C	simulated distillation
	water	H_2_0 + dissolved substances	gravimetric
solid	coke	burned-off substances	flue gas composition

Online analysis of oxygen, carbon monoxide, and carbon dioxide in the flue gas and the product gas was conducted using a unit that combines an infrared gas analyzer and a paramagnetic oxygen analyzer (NGA 2000 MLT 3, Emerson). The data of the flue gas measurements were used to calculate the coke production.

Furthermore, the concentrations of hydrocarbons and nitrogen in the product gas were analyzed in a gas chromatograph (GC-17A, Shimadzu). The GC utilizes two columns (Varian CP-Al_2_O_3_/Na_2_SO_4_; CP CarboPLOT P7), an FID, and a TCD.

The organic and the water phases (if present) were separated in a separation funnel, and both phases were weighted. The organic phase was filtered, and a simulated distillation (SimDist) of it was conducted utilizing a gas chromatograph (GC-17A from Shimadzu) with a Zebron ZB-1 column and an FID. The data are used to determine the boiling curve of the sample.

The organic phase was distilled in a laboratory distillation apparatus to obtain the gasoline lump. The cutoff temperature of 210 °C (final boiling point of gasoline according to standard DIN EN 228:2017-08) was chosen to achieve the maximum yield of gasoline, which is the main goal of the WASTE2ROAD project. The gasoline samples were sent to the OMV laboratory for further analysis. Additional analysis procedures were not disclosed for confidentiality purposes.

## Results and Discussion

The three different provided bio-liquids were all processable in the FCC pilot plant as co-feed with VGO in admixture of 95 wt % VGO and 5 wt % bio-liquid. The typical duration of continuous feeding in the course of an experiment with stable conditions is 2.5 h. This duration could not be reached with the experiment with 5 wt % PO, which was slightly unstable. However, this circumstance does not speak against the use of PO as a co-feed in an FCC unit. Pinho et al.^23^ elaborates why certain operational instabilities are inherent to small-scale units and can be avoided in commercial FCC units through structural measures, such as supplying oxygenated feeds via a feed line that is kept below 50 °C.

### FCC Product

**Figure 3:**
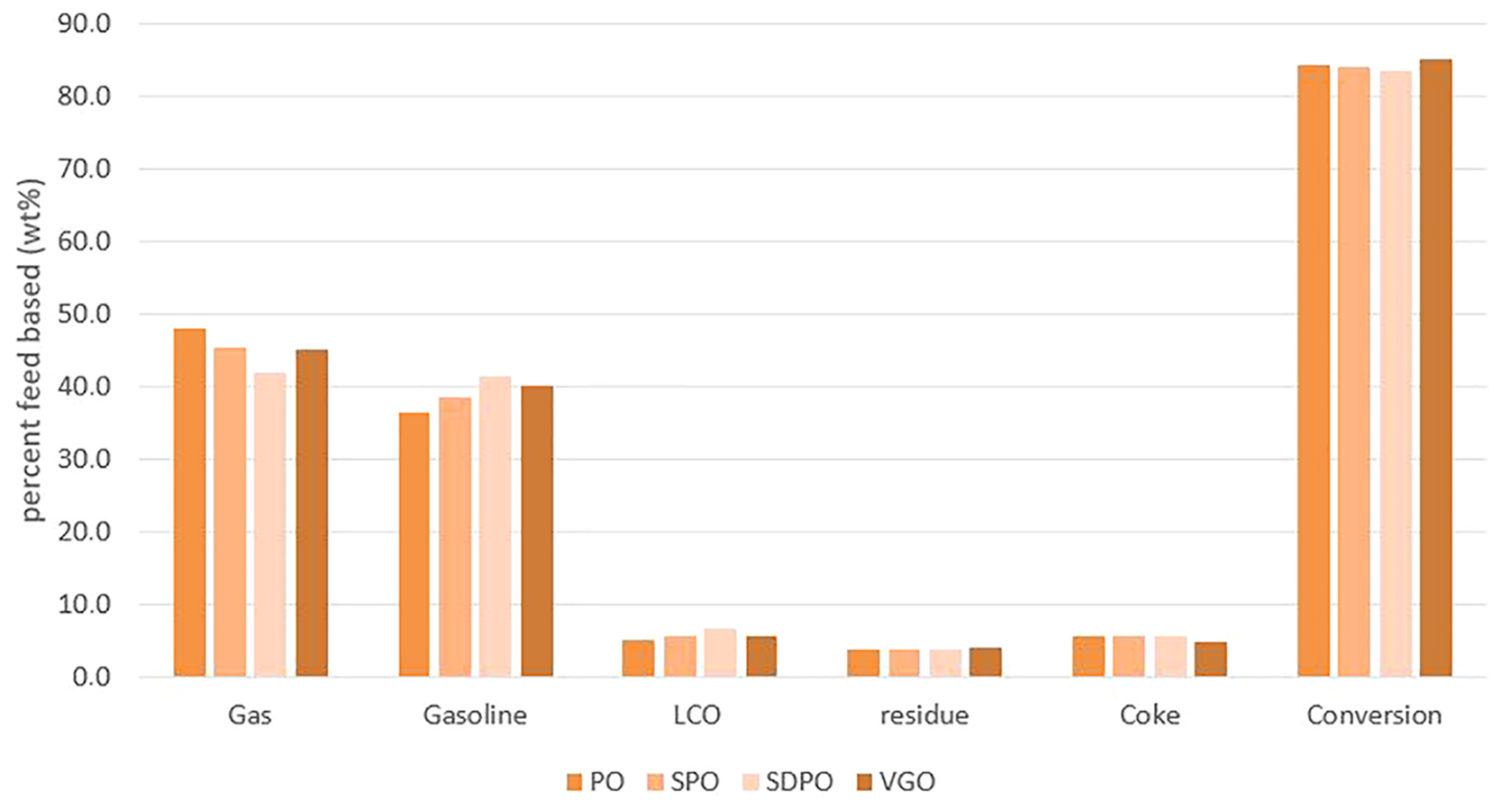
Product yields for catalytic cracking.

Figure 3 is a depiction of the values listed in Table 6 for the different lumps, with the exception of the lumps carbon oxides and water due to their small values.

**Table 6:** Feed Based Results of the Lump Model for the Different Experiments

	gas (wt %)	gasoline (wt %)	LCO (wt %)	residue (wt %)	coke (wt %)	carbon oxides (wt %)	water (wt %)	conversion (wt %)
PO	47.9	36.3	5.0	3.7	5.7	0.6	0.9	84.2
SPO	45.4	38.6	5.7	3.9	5.6	0.5	0.4	83.9
SDPO	42.0	41.5	6.8	3.9	5.6	0.3	0.0	83.5
VGO	45.0	40.0	5.8	4.0	4.9	0.3	0.0	85.0

The data show that a higher grade of treatment of the bio-liquid is accompanied by a decrease in gas production and a rise in gasoline production. The conversion is defined by the weight percentage of products (here the sum of gas and gasoline) based on the feed used and slightly decreases with the treatment grade of the bio-liquid. This is due to the fact that not only the gasoline lump increases with severity but also the LCO lump, which, in this work, is not taken into account as a product since it is partially cycled back into the process in the simulated plant. Overall, the addition of bio-liquid thus leads to a minor decrease of conversion in comparison to the use of pure VGO (∼1–1.5 wt %) and also to an increase of the produced amount of coke (∼1 wt %). Partially this is due to the lower amounts of moles of carbon that is fed into the reactor system.

The produced amount of water (Table 6) is 0.9 wt % for PO and 0.4 wt % for SPO. SDPO and VGO did not produce water. The collected water in the product is the sum of water present in the feed and water formed by the cracking reactions. Due to deoxygenation occurring during the cracking process not only in the form of dehydration but also as decarbonylation and decarboxylation,^24^ higher amounts of carbon oxides are seen in the experiments PO and SPO in comparison to SDPO and VGO.

#### Gasoline Lump

Table 7 shows the measured parameters of the gasoline samples that have set legal requirements in the standards ASTM D4814-16e^25^ or DIN EN 228:2017-08^26^ or both. The checkmark symbol means the parameter has to be measured but there is no value limitation. The values marked with an asterisk (*) were first published by Büchele et al.^27^

**Table 7:**
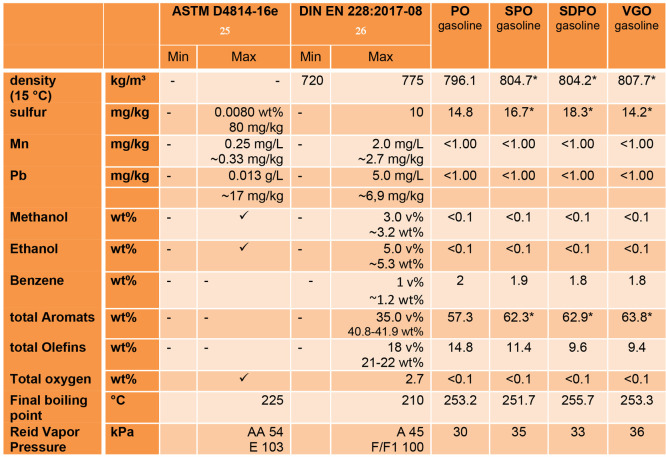
Measured Parameters of the Gasoline Samples with Legal Requirements in ASTM D4814-16e or DIN EN 228:2017-08^a^

a Checkmark means the parameter has to be measured but there is no value limitation; the values marked with an asterisk (*) were first published by Büchele et al.^27^.

The values from Pb and the total of olefins are below the set maximum values. The content of oxygenated compounds such as alcohols and ethers of all samples lie below 0.1 wt %. The lack of oxygenated components is due to the deoxygenation occurring during the cracking process. For Mn the value is below 2.0 mg/L, which is stated as maximum value in DIN EN 228:2017-08, but the limit of detection of the used analysis method is above the maximum value of 0.25 mg/L in ASTM D4814-16e.

The sulfur content of all samples is below the maximum value of 0.0080 wt % mentioned in ASTM D4814-16e, but above 10 mg/kg mentioned in DIN EN 228:2017-08. Sulfur content in FCC gasoline is strongly related to the sulfur content in the used feed. Between 2 and 20% of the initial sulfur end up in the FCC gasoline,^28^ and the content can be as high as 3000 mg/kg for gasoline from unhydrogenated VGO.^29^

The values for density (at 15 °C) and benzene and total aromatic content for all samples are above the maximum values defined in DIN EN 228:2017-08 (no values are stated in ASTM D4814-16e). Only the benzene value is slightly higher for the bio-liquid admixtures than for the pure VGO sample. The VGO gasoline samples have the highest values for density (15 °C) and total aromatic content. The vapor pressure of all gasoline samples is below the possible range between different fuel classes mentioned in the standards. The gasoline derived from PO has the lowest vapor pressure with 30 kPa, while VGO gasoline exhibits the highest vapor pressure with 36 kPa. All samples have a higher final boiling point than mentioned in the standards, but that is likely due to the distillation apparatus used in the present study, allowing higher-boiling compounds to be carried along. The utilized fractionating column between round-bottom flask and Liebig condenser was a 150 mm Vigreux column.

Since VGO gasoline is a gasoline blending component and the benzene value is only 0.2 wt % higher for the gasoline samples of the bio-liquid experiments, it can be concluded that the gasoline of all bio-liquid experiments can be used as gasoline blending component if the Mn value is low enough for ASTM D4814-16e. Higher sulfur values do not lead to exceeding the maximum sulfur content in the finished gasoline blend, since 80–95 wt % of the total sulfur content of the blend stems from FCC gasoline^28,30^ whereas FCC gasoline only makes up around 20–40% of the blend;^28,30,31^ thus the lower vapor pressure also does not pose a problem to reach the intended values in the blend.

Table 8 shows the measured parameters of the gasoline sample which must be measured according to ASTM D4814-16e, DIN EN 228:2017-08, or both. These parameters however have no indication of maximum or minimum values. The values marked with an asterisk (*) were first published by Büchele et al.^27^ The detailed breakdown for naphthenes, olefins, and paraffins is available in the Supporting Information.

**Table 8:**
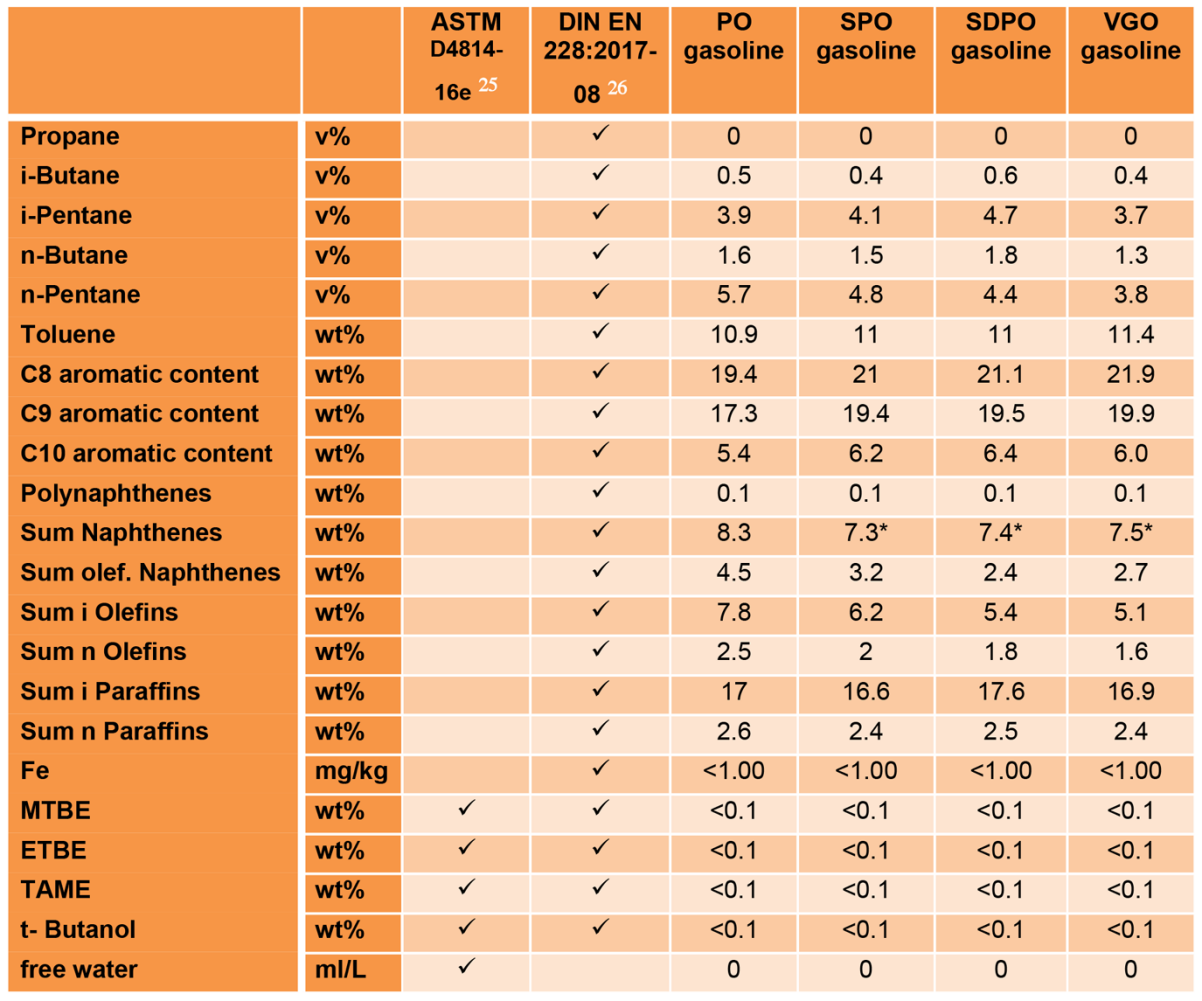
Measured Parameters of the Gasoline Samples That Have to Be Measured According to ASTM D4814-16e or DIN EN 228:2017-08 but Have No Indication of Maximum or Minimum Values^a^

a Checkmarks mean the parameter has to be measured according to this standard; the values marked with as asterisk (*) were first published by Büchele et al.^27^.

As listed in Table 7 the total aromatic content varies from 57.3 wt % (PO gasoline) to 63.8 wt % (VGO gasoline), with higher concentration of aromatics according to the treatment grade of the used bio-liquid. The main reason is the difference of the C8 and C9 aromatic contents (Table 8) between the PO gasoline and the VGO gasoline samples, which are both around 2.5 wt %, while differences between the other gasoline samples and VGO gasoline were lower.

The isopentane concentration of the gasoline increases with the treatment grade of the bio-liquid (3.9–4.7 wt %) and is higher than the pure VGO sample (3.7 wt %). The *n*-pentane concentration is also higher for the bio-liquid gasoline samples than for the pure VGO gasoline sample, but here the concentration decreases with the treatment grade of the bio-liquid.

The sum of olefinic naphthenes shows a big difference between PO gasoline (4.5 wt %), SPO gasoline (3.2 wt %), and VGO gasoline (2.7 wt %). SDPO gasoline has the lowest concentration with 2.4 wt %. The main differences are the concentrations of the C8, C7, and C6 olefinic naphthenes.

The sum of iso-olefins and the sum of *n*-olefins also show the same tendencies as the sum of olefinic naphthenes with the exception that the VGO gasoline sample has the lowest value and not the SDPO gasoline sample. The concentrations of iso-olefins are 7.8 wt % for PO gasoline, 6.2 wt % for SPO gasoline, 5.4 wt % for SDPO gasoline, and 5.1 wt % for VGO gasoline The main reason for the difference in the iso-olefin concentration are the iso- C6 and iso-C5 olefinic compounds. The concentrations of *n*-olefins are 2.5 wt % for PO gasoline, 2.0 wt % for SPO gasoline, 1.8 wt % for SDPO gasoline, and 1.6 wt % for VGO gasoline. The main reason for the difference in the *n*-olefin concentrations are the *n*-C5 and *n*-C4 olefinic compounds. The reduction of olefinic products with the severity of the hydrotreatment matches the findings of Fogassy et al.^15^ and Schuurman et al.^32^ and is caused by the consumption of hydrogen that is necessary for the dehydroxylation of the oxygenated components.

Table 9 shows the measured parameters of the gasoline samples that are not mentioned in ASTM D4814-16e or DIN EN 228:2017-08. The values marked with an astereisk (*) were first published by Büchele et al.^27^

**Table 9:** Measured Parameters of the Gasoline Samples That Are Not Mentiond in ASTM D4814-16e or DIN EN 228:2017-08^a^

		PO	SPO	SDPO	VGO
initial boiling point	°C	–5.2	–5.9	–5.4	–8.6
dissolved water	mg/kg	125*	184*	125*	101*
nitrogen	mg/kg	6.4	11.1*	7.5*	2.3*
hydrogen	wt %	12	11.8	11.9	11.8
carbon	wt %	87	87.2	87.1	87.3
Al	mg/kg	<1.00	<1.00	<1.00	<1.00
Ca	mg/kg	<1.00	<1.00	<1.00	<1.00
Cr	mg/kg	<1.00	<1.00	<1.00	<1.00
K	mg/kg	<1.00	<1.00	<1.00	<1.00
Mo	mg/kg	<1.00	<1.00	<1.00	<1.00
Na	mg/kg	<1.00	<1.00	<1.00	<1.00
Ni	mg/kg	<1.00	<1.00	<1.00	<1.00
Si	mg/kg	<1.00	<1.00	<1.00	<1.00
V	mg/kg	<1.00	<1.00	<1.00	<1.00
Zn	mg/kg	<1.00	<1.00	<1.00	<1.00
Hg	mg/kg	<0.003	<0.003	<0.003	<0.003
Ag	mg/kg	<1.00	<1.00	<1.00	<1.00
Cd	mg/kg	<1.00	<1.00	<1.00	<1.00
Cu	mg/kg	<1.00	<1.00	<1.00	<1.00
Mg	mg/kg	<1.00	<1.00	<1.00	<1.00
Sn	mg/kg	<1.00	<1.00	<1.00	<1.00
As	μg/kg	<10.0	<10.0	<10.0	<10.0
sum of halogens (Cl, Br) as Cl	mg/kg	<1	<1	<1	<1

a The values marked with an asterisk (*) were first published by Büchele et al.^27^.

The amount of dissolved water in the gasoline sample is the lowest for the VGO gasoline (101 mg/kg) and the highest for the SPO gasoline (184 mg/kg). The nitrogen concentrations of the gasoline samples are 2.3 mg/kg for VGO gasoline, 6.4 mg/kg for PO gasoline, 7.5 mg/kg for SDPO gasoline, and 11.1 mg/kg. The carbon and hydrogen contents only vary between 0.3 wt % points. All of the additionally measured element concentrations are below the detection limit of the respective methods used.

## Conclusion

The higher grade of treatment of bio-liquid is accompanied by a decrease in gas production and a rise in gasoline production during the FCC process. Overall, the addition of bio-liquid leads to a minor decrease of conversion, which is the weight percentage of product (gas and gasoline) based on the feed used, in comparison to the use of pure VGO (∼1–1.5 wt %) and to an increase of the amount of coke (∼1 wt %) produced.

Since FCC gasoline from VGO is a currently used gasoline blending component, it can be concluded that the gasoline of all bio-liquid experiments can be used as a gasoline blending component if the Mn value is low enough for ASTM D4814-16e, the higher sulfur values do not lead to exceeding the maximum value of sulfur in the finished gasoline blend, and the lower vapor pressure does not pose a problem to reach the intended values in the blend.

Not all key parameters of gasoline could be checked due to the amount of gasoline samples available. However, it shows the potentiality of the fluid catalytic cracking process as a possible refinement method for bio-liquids to enable higher biofuel content in commercially available fuel.

## Supporting Information Available

Detailed breakdown of the paraffins, olefins, and naphthenes present in the gasoline samples (Table S1) (PDF)
