# Supplementary material for: Wood Derived Fast Pyrolysis Bio-liquids as Co-feed in a Fluid Catalytic Cracking Pilot Plant: Effect of Hydrotreatment on Process Performance and Gasoline Quality
Source: Energy Fuels. 2022 Aug 10;36(17):10243–50. doi: 10.1021/acs.energyfuels.2c01736 (PMC9442577; doi:10.1021/acs.energyfuels.2c01736)
Supplement: Supplementary file 1 — ef2c01736_si_001.pdf [file ef2c01736_si_001.pdf]

# Wood derived fast pyrolysis bio-liquids as co-feed in a fluid catalytic cracking pilot plant: effect of hydrotreatment on process performance and gasoline quality

*Helene Lutz [1,*], Marco Büchele [1], Florian Knaus [1], Alexander Reichhold [1], Wolfgang Vollenhofer[2], Robbie Venderbosch[3]*

1) Institute of Environmental, Chemical and Bioscience Engineering (ICEBE), Technische Universität Wien, Austria; e-mail: Helene.Lutz@tuwien.ac.at

2) OMV Refining & Marketing AG, Trabrennstraße 6-8, 1020 Vienna, Austria

3) BTG Biomass technology Group B.V., Enschede, 7521, The Netherlands

*Table S1: Addition to parameters of the gasoline samples that have to be measured according to ASTM D4814-16e or DIN EN 228:2017-08 but have no indication of maximum or minimum values; ✓ means the parameter has to be measured according to this standard; the values marked with * were first published by Büchele et al. [27]*

| | | ASTM D4814-16e [25] | DIN EN 228:2017-08 [26] | PO gasoline | SPO gasoline | SDPO gasoline | VGO gasoline |
|---|---|---|---|---|---|---|---|
| **Propane** | **v%** | | ✓ | 0 | 0 | 0 | 0 |
| **i-Butane** | **v%** | | ✓ | 0.5 | 0.4 | 0.6 | 0.4 |
| **i-Pentane** | **v%** | | ✓ | 3.9 | 4.1 | 4.7 | 3.7 |
| **n-Butane** | **v%** | | ✓ | 1.6 | 1.5 | 1.8 | 1.3 |
| **n-Pentane** | **v%** | | ✓ | 5.7 | 4.8 | 4.4 | 3.8 |
| **Toluene** | **wt%** | | ✓ | 10.9 | 11 | 11 | 11.4 |
| **C8 aromatic content** | **wt%** | | ✓ | 19.4 | 21 | 21.1 | 21.9 |
| **C9 aromatic content** | **wt%** | | ✓ | 17.3 | 19.4 | 19.5 | 19.9 |
| **C10 aromatic content** | **wt%** | | ✓ | 5.4 | 6.2 | 6.4 | 6.0 |
| **C5 Naphthenes** | **wt%** | | ✓ | 0.2 | 0.2 | 0.2 | 0.2 |

| | | | | | | | |
|---|---|---|---|---|---|---|---|
| C6 Naphthenes | wt% | | ✓ | 2 | 1.8 | 1.8 | 1.7 |
| C7 Naphthenes | wt% | | ✓ | 2.8 | 2.5 | 2.5 | 2.7 |
| C8 Naphthenes | wt% | | ✓ | 1.9 | 1.6 | 1.7 | 1.7 |
| C9 Naphthenes | wt% | | ✓ | 1 | 0.8 | 0.9 | 0.9 |
| C10 Naphthenes | wt% | | ✓ | 0.4 | 0.3 | 0.3 | 0.3 |
| C11 Naphthenes | wt% | | ✓ | 0 | 0 | 0 | 0.0 |
| Polynaphthenes | wt% | | ✓ | 0.1 | 0.1 | 0.1 | 0.1 |
| Sum Naphthenes | wt% | | ✓ | 8.3 | 7.3* | 7.4* | 7.5* |
| C5 olef. Naphthenes | wt% | | ✓ | 0.3 | 0.2 | 0.2 | 0.2 |
| C6 olef. Naphthenes | wt% | | ✓ | 1.4 | 1 | 0.8 | 0.8 |
| C7 olef. Naphthenes | wt% | | ✓ | 1.7 | 1.2 | 0.9 | 1.1 |
| C8 olef. Naphthenes | wt% | | ✓ | 0.9 | 0.6 | 0.5 | 0.5 |
| C9 olef. Naphthenes | wt% | | ✓ | 0.3 | 0.2 | 0.1 | 0.1 |
| C10 olef. Naphthenes | wt% | | ✓ | 0 | 0 | 0 | 0 |
| C11 olef. Naphthenes | wt% | | ✓ | 0 | 0 | 0 | 0 |
| Sum olef. Naphthenes | wt% | | ✓ | 4.5 | 3.2 | 2.4 | 2.7 |
| i C4 Olefins | wt% | | ✓ | 0.3 | 0.3 | 0.3 | 0.2 |
| i C5 Olefins | wt% | | ✓ | 3.3 | 2.8 | 2.5 | 2.1 |
| i C6 Olefins | wt% | | ✓ | 2.5 | 1.9 | 1.5 | 1.6 |
| i C7 Olefins | wt% | | ✓ | 1 | 0.7 | 0.6 | 0.7 |
| i C8 Olefins | wt% | | ✓ | 0.4 | 0.3 | 0.2 | 0.3 |
| i C9 Olefins | wt% | | ✓ | 0.2 | 0.1 | 0.1 | 0.1 |
| i C10 Olefins | wt% | | ✓ | 0.2 | 0.2 | 0.2 | 0.2 |
| i C11 Olefins | wt% | | ✓ | 0 | 0 | 0 | 0.0 |
| n C3 Olefins | wt% | | ✓ | 0 | 0 | 0 | 0.0 |
| n C4 Olefins | wt% | | ✓ | 1.5 | 1.3 | 1.2 | 1.0 |
| n C5 Olefins | wt% | | ✓ | 0.8 | 0.6 | 0.5 | 0.5 |
| n C6 Olefins | wt% | | ✓ | 0.2 | 0.1 | 0.1 | 0.1 |
| n C7 Olefins | wt% | | ✓ | 0 | 0 | 0 | 0.0 |
| n C8 Olefins | wt% | | ✓ | 0 | 0 | 0 | 0.0 |
| n C9 Olefins | wt% | | ✓ | 0 | 0 | 0 | 0.0 |
| n C10 Olefins | wt% | | ✓ | 0 | 0 | 0 | 0.0 |
| n C11 Olefins | wt% | | ✓ | 0 | 0 | 0 | 0.0 |
| Sum i Olefins | wt% | | ✓ | 7.8 | 6.2 | 5.4 | 5.1 |
| Sum n Olefins | wt% | | ✓ | 2.5 | 2 | 1.8 | 1.6 |
| i C4 Paraffins | wt% | | ✓ | 0.3 | 0.3 | 0.4 | 0.3 |
| i C5 Paraffins | wt% | | ✓ | 3.3 | 3.5 | 4.1 | 3.2 |
| i C6 Paraffins | wt% | | ✓ | 4.9 | 4.8 | 4.9 | 4.6 |
| i C7 Paraffins | wt% | | ✓ | 2.8 | 2.7 | 2.7 | 2.9 |
| i C8 Paraffins | wt% | | ✓ | 1.9 | 1.9 | 1.9 | 2.1 |
| i C9 Paraffins | wt% | | ✓ | 1.3 | 1.3 | 1.3 | 1.5 |
| i C10 Paraffins | wt% | | ✓ | 0.7 | 0.6 | 0.7 | 0.7 |
| i C11 Paraffins | wt% | | ✓ | 1.8 | 1.6 | 1.5 | 1.6 |

| | | | | | | | |
|---|---|---|---|---|---|---|---|
| **n C3 Paraffins** | **wt%** | | ✓ | 0 | 0 | 0 | 0.0 |
| **n C4 Paraffins** | **wt%** | | ✓ | 0.1 | 0.1 | 0.1 | 0.1 |
| **n C5 Paraffins** | **wt%** | | ✓ | 0.5 | 0.5 | 0.5 | 0.5 |
| **n C6 Paraffins** | **wt%** | | ✓ | 0.6 | 0.5 | 0.5 | 0.5 |
| **n C7 Paraffins** | **wt%** | | ✓ | 0.5 | 0.3 | 0.4 | 0.4 |
| **n C8 Paraffins** | **wt%** | | ✓ | 0.3 | 0.3 | 0.3 | 0.3 |
| **n C9 Paraffins** | **wt%** | | ✓ | 0.2 | 0.2 | 0.3 | 0.2 |
| **n C10 Paraffins** | **wt%** | | ✓ | 0.5 | 0.5 | 0.5 | 0.5 |
| **n C11 Paraffins** | **wt%** | | ✓ | 0 | 0 | 0 | 0.0 |
| **Sum i Paraffins** | **wt%** | | ✓ | 17 | 16.6 | 17.6 | 16.9 |
| **Sum n Paraffins** | **wt%** | | ✓ | 2.6 | 2.4 | 2.5 | 2.4 |
| **Fe** | **mg/kg** | | ✓ | <1.00 | <1.00 | <1.00 | <1.00 |
| **MTBE** | **wt%** | ✓ | ✓ | <0.1 | <0.1 | <0.1 | <0.1 |
| **ETBE** | **wt%** | ✓ | ✓ | <0.1 | <0.1 | <0.1 | <0.1 |
| **TAME** | **wt%** | ✓ | ✓ | <0.1 | <0.1 | <0.1 | <0.1 |
| **t- Butanol** | **wt%** | ✓ | ✓ | <0.1 | <0.1 | <0.1 | <0.1 |
| **free water** | **ml/L** | ✓ | | 0 | 0 | 0 | 0 |
